# The Antimicrobial Activity of *Annona emarginata* (Schltdl.) H. Rainer and Most Active Isolated Compounds against Clinically Important Bacteria

**DOI:** 10.3390/molecules23051187

**Published:** 2018-05-16

**Authors:** Juan G. Dolab, Beatriz Lima, Ewelina Spaczynska, Jiri Kos, Natividad H. Cano, Gabriela Feresin, Alejandro Tapia, Francisco Garibotto, Elisa Petenatti, Monica Olivella, Robert Musiol, Josef Jampilek, Ricardo D. Enriz

**Affiliations:** 1 Faculty of Chemistry Biochemistry and Pharmacy, National University of San Luis, Chacabuco 917, 5700 San Luis, Argentina; juandolab@gmail.com (J.G.D.); fgaribotto@gmail.com (F.G.); elipete@unsl.edu.ar (E.P.); olivellamonica@gmail.com (M.O.); 2 Institute of Biotechnology-Institute of Basic Sciences, National University of San Juan, Av. Libertador General San Martin 1109, 5400 San Juan, Argentina; blima@unsj.edu.ar (B.L.); natividadhc17@hotmail.com (N.H.C.); gferesin@unsj.edu.ar (G.F.); atapia@unsj.edu.ar (A.T.); 3 Institute of Chemistry, University of Silesia, 75 Pulku Piechoty 1, 41500 Chorzow, Poland; ewelina.spaczynska@gmail.com (E.S.); robert.musiol@us.edu.pl (R.M.); 4 Department of Pharmaceutical Chemistry, Faculty of Pharmacy, Comenius University, Odbojarov 10, 83232 Bratislava, Slovakia; jirikos85@gmail.com; 5 Multidisciplinary Institute of Biological Research IMIBIO-CONICET, Chacabuco 917, 5700 San Luis, Argentina

**Keywords:** *Annona emarginata*, (*R*)-2-(4-methylcyclohex-3-en-1-yl)propan-2-yl (*E*)-3-(4-hydroxyphenyl)-acrylate, antibacterial activity, *Staphylococcus aureus*, structure-activity relationships

## Abstract

*Annona emarginata* (Schltdl.) H. Rainer, commonly known as “arachichú”, “araticú”, “aratigú”, and “yerba mora”, is a plant that grows in Argentina. Infusions and decoctions are used in folk medicine as a gargle against throat pain and for calming toothache; another way to use the plant for these purposes is chewing its leaves. Extracts from bark, flowers, leaves, and fruits from *A. emarginata* were subjected to antibacterial assays against a panel of Gram (+) and Gram (−) pathogenic bacteria according to Clinical and Laboratory Standards Institute protocols. Extracts from the stem bark and leaves showed moderate activity against the bacteria tested with values between 250–1000 µg/mL. Regarding flower extracts, less polar extracts (hexane, dichloromethane) showed very strong antibacterial activity against methicillin-sensitive *Staphylococcus aureus* ATCC 25923 and methicillin-resistant *S. aureus* ATCC 43300 with values between 16–125 µg/mL. Additionally, hexane extract showed activity against *Klebsiella pneumoniae* (MIC = 250 µg/mL). The global methanolic extract of the fruits (MeOHGEF) was also active against the three strains mentioned above, with MICs values 250–500 µg/mL. Bioassay-guided fractionation of MeOHGEF led to the isolation of a new main compound—(*R*)-2-(4-methylcyclohex-3-en-1-yl)propan-2-yl (*E*)-3-(4-hydroxyphenyl)acrylate (**1**). The structure and relative configurations have been determined by means of 1D and 2D NMR techniques, including COSY, HMQC, HMBC, and NOESY correlations. Compound 1 showed strong antimicrobial activity against all Gram (+) species tested (MICs = 3.12–6.25 µg/mL). In addition, the synthesis and antibacterial activity of some compounds structurally related to compound **1** (including four new compounds) are reported. A SAR study for these compounds was performed based on the results obtained by using molecular calculations.

## 1. Introduction

Some genera from the family *Annonaceae* are an important natural source of products with antifungal [1], bactericidal [2], pesticidal [3], herbicidal [4], antimalarial [5], antiprotozoal [6], and cytostatic [7,8] activity, especially some chemically isolated components (primarily from seeds, leaves, and bark). These compounds include flavonoids, alkaloids, and acetogenins. This family is widely distributed in tropical and subtropical regions around the world in diversity of habitats (forests, scrub, and grassland), with higher prevalence in rain forests. In general, representatives of the family are shrubs, but it is also possible to find dwarf shrubs or small trees and, rarely, canopy trees or lianas among them. The Annona genus comprises ca. 160 species from the tropical and subtropical areas, from both hemispheres. Four species grow mainly in Argentina: *Annona emarginata* (Schltdl.) H. Rainer (=*Rollinia emarginata* Schltdl.), *A. neosalicifolia* H. Rainer (=*Rollinia salicifolia* Schltdl.), *A. nutans* (R.E. Fr.), and *A. rugulosa* (Schltdl.) H. Rainer. Previous studies related to ethnopharmacological use have shown that the infusions and decoctions of the leaves of *A. emarginata*, commonly known as “arachichú”, “araticú”, “aratigú”, and “yerba mora”, are used in folk medicine as a gargle against throat pain and for calming the toothache. Another way it is used is by absorbing juice produced by chewing the leaves [9,10]. Antiprotozoal [11] and antifeedant [12] properties have been reported. Regarding its chemical composition, rolliniastatin-1, sylvaticin, squamocin, rollidecin B, lirioresinol B, liriodenine [11]; vomifoliol, dehydrovomifoliol, blumenol C, loliolide, 7-epiloliolide, vanillin, dihydroactinolide [12]; ursolic acid, oleanolic acid, 8-*trans*-*p*-coumaroyloxy-α-terpineol, quercetin-3-*O*-α-l-arabinopyranosyl(1→2)-α-l-rhamnopyranoside [13]; alkaloids including two aporphines (−)-anonaine, (−)-asimilobine and one benzyltetrahydroisoquinoline, (+)-reticuline [14], and cyanogenic glycosides [15] have been previously isolated from its aerial parts. Resistance to antimicrobials is a problem that increasingly threatens the world population [16]. The World Health Organization established as “priority pathogens” those resistant to antibiotics, which include 12 families of bacteria most dangerous to human health, the so-called “superbugs”. Among them, especially the threat posed by Gram (−) bacteria resistant to multiple antibiotics is highlighted. Among the Gram (+) bacteria, methicillin-resistant *Staphylococcus aureus* (MRSA) [17] is one of the most important. Thus, the development of new molecules, alternative strategies, and prevention and control measures is necessary to avoid this problem.

The main objective of this work is to determine the antibacterial effect of different extracts of *A. emarginata* (Schltdl.) H. Rainer, which is widely used in folk medicine in the northeast part of Argentine. Another goal is to try to determine which active compound is responsible for such activity. Thus, this work has been done in several stages. In the first step, the antimicrobial properties of extracts of *A. emarginata* were studied, and the bioassay-guided isolation of its most active compound was made. The compound was tested against an extended panel of both standardized and clinical opportunistic pathogenic bacteria. The species were selected to include Gram (+) and Gram (−) species of the most common human pathogens. Once the structure of the compound responsible for the antibacterial activity was determined, some structurally related compounds were synthesized. Finally, based on molecular calculations, we performed a study of the structure-activity relationships of antibacterial compounds of the different series reported here.

## 2. Results and Discussion

### 2.1. Antimicrobial Evaluation of A. emarginata Extracts and Its Active Ingredient

The extracts from the stem bark and leaves of *A. emarginata* showed moderate activity against the bacteria tested, with values between 250 and 1000 µg/mL (Table 1). Regarding flower extracts, the less polar (hexane, dichloromethane) extracts, HFlE and DCMFlE, showed very strong antibacterial activity against methicillin-sensitive *S. aureus* (MSSA) ATCC 25923 and methicillin-resistant *S. aureus* (MRSA) ATCC 43300, with values between 16 and 125 µg/mL. Additionally, hexane extract showed activity against *Klebsiella pneumoniae* (MIC = 250 µg/mL). The global methanolic extract from the fruits (MeOHGFE) was also active against the three strains mentioned above, with minimum inhibitory concentration (MIC) values between 250–500 µg/mL. In order to identify bioactive compounds, a bioassay-guided fractionation of the MeOHGFE was performed.

**Figure 1:**
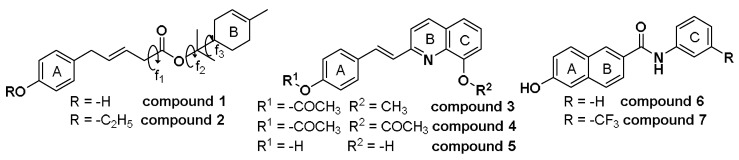
Structural features of compounds studied. Torsional angles are shown for compound **1**.

Table 2 and Table 3 show the antimicrobial activity of fractions and subfractions of the MeOHGFE. Fraction F_3_ displayed the strongest antibacterial activity (Table 2) against MSSA, MRSA, *Escherichia coli* ATCC 25922 (MIC = 500 µg/mL), and *K. pneumoniae* (MIC = 125 µg/mL). To gain further information about their activity spectrum against Gram (+) strains, we added a panel of clinical isolates obtained from human patients (coagulase negative *Staphylococcus aureus* 502, *Streptococcus pyogenes* and *Streptococcus agalactiae*) (Table 3 and Table 4). Fraction F_3_ was further percolated on Sephadex LH-20 affording 5 subfractions (I–V). One of them, subfraction F_IV_, showed strong antibacterial activity with MIC = 16 µg/mL against all the Gram (+) bacteria tested, except for coagulase negative *S. aureus* 502 (MIC = 31.2 µg/mL), *Escherichia coli* LM2 (MIC = 125 µg/mL), and *K. pneumoniae* (MIC = 250 µg/mL, respectively). On the basis of the previous results, subfraction F_IV_ was successively purified by Sephadex LH-20 and silica gel column, affording the main antibacterial compound **1** (Figure 1). As we expected, compound **1** showed stronger antimicrobial activity against both Gram (+) ATCC strains with MICs = 3.12–6.25 µg/mL, and the expanded panel strains were also sensitive to this compound with MICs value equal 3.12 µg/mL (Table 4). In contrast, compound **2** (see below) was practically inactive.

### 2.2. Natural Lead Structure

**Figure 2:**
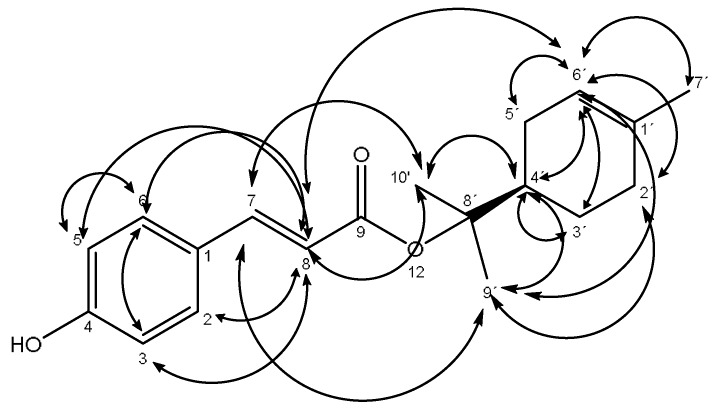
Key NOESY correlations of compound **1**.

The strongest antimicrobial subfraction IV was successively chromatographed on silica gel to afford pure compound **1**, which was identified by its spectroscopic data, including ^1^H, ^13^C-NMR, and HR-EIMS spectra as (*R*)-2-(4-methylcyclohex-3-en-1-yl)propan-2-yl (*E*)-3-(4-hydroxyphenyl)acrylate (**1**), see Figure 1 and spectra in Supplementary Material. The relevant NOESY correlations (Figure 2), a strong positive Cotton effect observed in the CD spectrum and spectroscopic data, which are in partial agreement with those previously published for the compound 9-*trans*-*p*-coumaroyloxy-α-terpineol, isolated from *Haplopappus taeda* [18], support the structure and absolute configuration proposed in Figure 2. In addition, (DFT) (B3LYP/6-31G (d)) calculations suggest that this structure is 0.46 Kcal/mol more stable than its isomeric form, giving additional support to the experimental data.

### 2.3. Synthetic Analogues

Observing the structure of compound **1**, it is interesting to note that the moiety having the aromatic ring corresponds to a structure very similar to that of *p*-coumaric and caffeic acids. Previously, it was reported that analogues of *p*-coumaric and caffeic acids have significant antibacterial and antifungal activities [19,20]. In fact, several phenolic compounds and derivatives possessing antifungal effects have been reported. For example, gallic acid has been reported to prevent aflatoxin biosynthesis by *Aspergillus flavus* [21]. Two compounds of natural origin, 1-(3′-methoxypropanoyl)-2,4,5-trimethoxybenzene and 2-(2*Z*)-(3-hydroxy-3,7-dimethylocta-2,6-dienyl)-1,4-benzenediol, isolated from the root bark of *Cordia alliodora,* have been also reported as antifungal and larvicidal compounds [22]. In turn, Kim et al. [23] reported antifungal effects of several phenolic compounds, including cinnamic, *m*-coumaric, *p*-coumaric, and caffeic acids; therefore, the significant antibacterial activity found for compound **1** should not be so surprising.

**Scheme 1:**
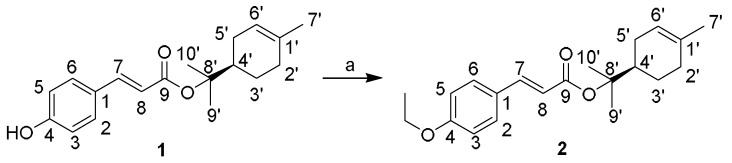
(*R*)-2-(4-Methylcyclohex-3-en-1-yl)propan-2-yl (*E*)-3-(4-ethoxyphenyl)acrylate (**2**)—etherified product of (*R*)-2-(4-methylcyclohex-3-en-1-yl)propan-2-yl (*E*)-3-(4-hydroxyphenyl)-acrylate (**1**). *Reagents and conditions*: (a) *t*-BuOK, DMSO, CH_3_CH_2_I, 30 min.

At this stage of the study, efforts were focused on finding new compounds structurally related to lead compound **1** that may have antibacterial activity. First, (*R*)-2-(4-methylcyclohex-3-en-1-yl)-propan-2-yl (*E*)-3-(4-ethoxyphenyl)acrylate (**2**) was obtained by a simple modification of compound **1**, see Scheme 1. Interestingly, compound **2** was devoid of antibacterial activity displaying MICs values ˃50 µg/mL. It should be noted that the only structural difference between compounds **1** and **2** is the ethylation of the hydroxyl group of compound **1**.

**Scheme 2:**
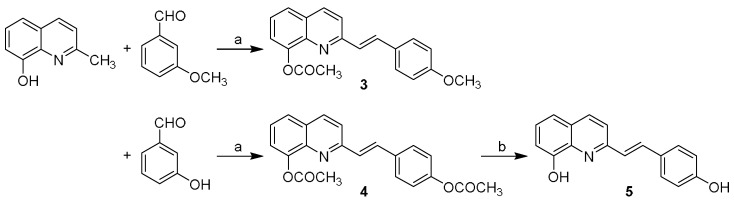
Synthesis of compound **3**–**5**. *Reagents and conditions*: (a) Ac_2_O, 80% AcOH, 18 h at 130 °C, and N_2_ atmosphere; (b) MeOH, K_2_CO_3_, and HCl.

Then, compounds **3**–**5** were synthesized, in which the first moiety of compound **1** was maintained, while the second part (4-hydroxyphenyl) of the molecule was replaced by a fused ring (rings A and B) of the quinoline scaffold (see Figure 1). Styrylquinoline derivatives **3** and **4** were prepared by condensation of the quinoline derivative with an appropriate aldehyde in a mixture of acetic anhydride/acetic acid. 2-[2-(4-Hydroxyphenyl)vinyl]quinolin-8-ol (**5**) was hydrolysed in methanol with K_2_CO_3_, then precipitated by concentrated HCl and crystallized from ethanol, see Scheme 2.

**Figure 3:**
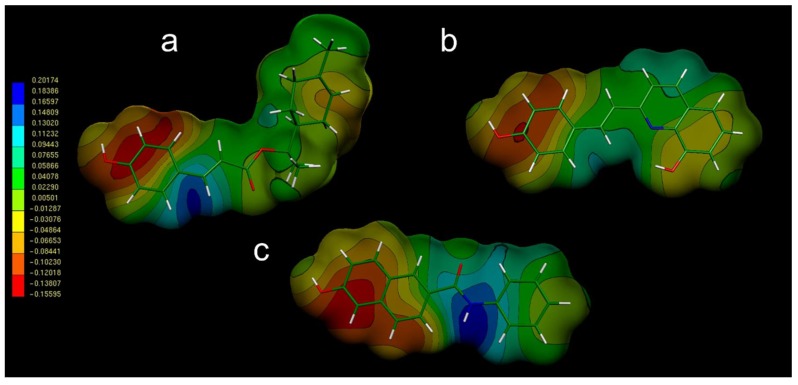
Electrostatic potential-encoded electron density surfaces of core structures of compounds **1** (**a**), **3** (**b**), and **6** (**c**). The colouring represents electrostatic potential, with red indicating the strongest attraction to a positive point charge and blue indicating the strongest repulsion. The electrostatic potential is the energy of interaction of the positive point charge with the nuclei and electrons of a molecule. It provides a representative measure of overall molecular charge distribution.

After synthesis of compounds **3**–**5**, their potential antibacterial activities were evaluated. While compound **5** showed moderate antibacterial activity, compounds **3** and **4** were inactive. It is interesting to note that the only structural difference between compound **5** and compounds **3** and **4** is the replacement of the OH group by OAc and OCH_3_, respectively. These results are in accord with those obtained for compounds **1** and **2**, since the ethylation of the OH group of **1** produced significant loss of antibacterial activity. From these results, it appears that the presence of a proton donor group in the ring A of these compounds could play an important role in the antibacterial effect, e.g., compare Gonec et al. [24] with Kos et al. [25,26]. In order to understand better this behaviour, a conformational and electronic study was performed for these compounds with the aim to determine their stereo-electronic characteristics. Details about the type of molecular calculations and the methodology used are explained in the Section 3.4. In addition, details about the conformational analysis of the compounds are also given in the same section. Knowledge of the stereo-electronic attributes and properties of these compounds will contribute significantly to the elucidation of the structural requirements to produce antibacterial activity. Molecular electrostatic potentials (MEP) are of particular value, because they allow the visualization and assessment of the capacity of a molecule to interact electrostatically with a putative-binding site [27,28,29]. Thus, once the conformational behaviours of compounds reported here had been analysed, an electronic study of their respective preferred conformations by using Electrostatic Potentials Maps (EPMs) was performed. Figure 3 gives the Molecular Electrostatic Potentials (MEP) obtained for compounds **1** and **3**. The MEP obtained for compound **1** (Figure 3a) exhibit three characteristic regions: a clear minimum value (deep red zone with V(r)min ≈ −0.1559 el/au3) in the vicinity of the OH group of the ring A, a positive region (deep blue zone with V(r)max ≈ 0.20 el/au3) located in the linker chain, and an extended hydrophobic zone (deep and light green zone with an almost neutral potential V(r)med ≈ −0.0011 el/au3) in the zone near to the ring B.

It is interesting to note that the MEP of compound **3** (Figure 3b) is similar to that obtained for **1**. However, for compound **3**, both the most electronegative zone (zone with red and orange tones) and the electropositive part of the molecule (area with bluish tonalities) are significantly more tenuous with values of V(r)min ≈ −0.120 el/au3 and V(r)max ≈ 0.110 el/au3, respectively. These results are in agreement with the experimental data, considering that compound **3** displayed lower antibacterial activity than compound **1**.

**Scheme 3:**
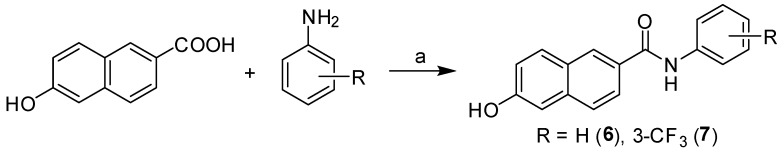
Synthesis of ring-substituted 6-hydroxynaphthalene-2-carboxanilides **6** and **7**. *Reagents and conditions*: (a) PCl_3_, chlorobenzene, and microwave irradiation (MW) [25].

A series of carboxanilides with antimycobacterial properties, including 6-hydroxy-*N*-phenylnaphthalene-2-carboxamide (**6**) and 6-hydroxy-*N*-(3-trifluoromethylphenyl) naphthalene-2-carboxamide (**7**) (see Figure 1), was reported recently [25]. They were prepared according to Scheme 3 by modified microwave-assisted synthesis in one step with excellent yields. At first, the carboxyl group was activated with phosphorus trichloride. The final amide was immediately formed by aminolysis of acyl chloride by a ring-substituted aniline in dry chlorobenzene. All the crude target compounds were recrystallized from ethanol.

It should be noted that these compounds are naphthalene analogues (carbon isosteres) of the quinoline scaffold, i.e., they also possess fused rings in their structure (rings A and B), which gives a certain structural similarity to the first portion of compound **1**. Next, a conformational and electronic study of compounds **6** and **7** was performed, and the results were compared with those obtained for the rest of compounds studied here. Figure 3c indicates that compound **6** displays MEP that are very similar to those obtained for compound **1** (compare Figure 3a,c). In this case, the values obtained for V(r)max and V(r)min are very similar to those observed in compound **1**. A very similar result was obtained for compound **7**. Based on these results, it was decided to test these compounds as potential antibacterial agents. Compounds **6** and **7**, especially compound **7**, showed significant antibacterial effects against the different strains studied here (Table 4). These experimental results fully agree with the molecular calculations and give additional support to the proposed pharmacophoric pattern. Once again, the presence of a proton donor group at ring A appears to be an essential structural requirement for antibacterial effect.

## 3. Materials and Methods

### 3.1. Extractions and Isolation

#### 3.1.1. General Methods

^1^H and ^13^C-NMR spectra were recorded on a High Resolution Spectrometer Advance 400 (working frequency 400 MHz and 100 MHz, respectively) at ambient temperature in CDC_l3_ (Aldrich, St. Louis, MO, USA). Carbon spectra assignments are supported by DEPT-135 spectra, ^13^C-^1^H (HMQC) and ^13^C-^1^H (HMBC), and NOESY correlations. High-resolution mass spectrums were measured on Bruker Micro TOF Q II equipment (Bruker, Karlsruhe, Germany), operated with an ESI source in the positive mode. Optical rotations were measured with a Jasco polarimeter DIP-1000 P-1010 34 (Jasco, Easton, MD, USA). The CD (Circular Dichroism) and UV-VIS spectra of methanol solutions of **1** and **2** were recorded simultaneously using a Jasco J-810 spectropolarimeter, model J-810 150S and a 100 lm quartz cuvette with resolution 1 nm, time constant 1 s, scan speed 100 nm/min, and 3 accumulations, employing standard sensitivity and a measurement range of 700–190 nm.

#### 3.1.2. Plant Material

The aerial parts (leaves, flowers, and fruits) of *A. emarginata* were collected in San Cosme, province of Corrientes (Argentina) at 27.36667 lat. S; 58.51667 long. W, in the summer of 2013 and were authenticated by Dra. Elisa Petenatti and L.A. Del Vitto. A voucher specimen (L.A. Del Vitto & E.M. Petenatti # 9490) has been deposited at the Herbarium, National University of San Luis, Argentina.

#### 3.1.3. Preparation of Extracts

Finely ground *A. emarginata* fruits (100 g) were extracted at room temperature using methanol MeOH (200 mL × 3-fold × 96 h each), filtered and concentrated under reduced pressure to afford the corresponding crude global methanolic extract (MeOHGFE; 17.7 g, yield 17.7% *w*/*w*). Dried and finely ground *A. emarginata* flowers (100 g) were successively extracted at room temperature (200 mL × 3-fold × 96 h, each 3 × 24 h) with hexane (H), dichloromethane (DCM), ethyl acetate (EtOAc), and methanol (MeOH). Then, solvents were evaporated under vacuum to give flower extracts HFlE, DCMFlE, EtOAcFlE, and MeOHFlE with yields (*w*/*w*) of 4.3%, 1.9%, 0.66%, and 5.9%, respectively. Finely ground dried *A. emarginata* stem bark (100 g) was successively extracted at room temperature (200 mL × 3-fold × 96 h, each 3 × 24 h) with H, DCM, EtOAc, and MeOH. The solvents were evaporated under vacuum to afford bark extracts HBE, DCMBE, EtOAcBE, and MeOHBE with yields (*w*/*w*) of 0.92%, 0.27%, 0.34%, and 4.23%, respectively. Finely ground dried *A. emarginata* leaves (100 g) were successively extracted at room temperature (200 mL × 3-fold × 96 h, each 3 × 24 h) with H, DCM, EtOAc, and MeOH. Then, solvents were evaporated under vacuum to produce leaf extracts HLE, DCMLE, EtOAcLE, and MeOHLE, affording 0.63%, 1.54%, 0.75%, and 1.02% yield (*w*/*w*), respectively.

#### 3.1.4. Bioassay-Guided Fractionation of *Annona emarginata* Fruit Extracts (MeOHGFE)

MeOHGFE (11g) was applied onto a column (column length 70 cm, i.d. 5 cm) containing silica gel (0.063-0.2 mesh, Merck 60, Merck, Darmstadt, Germany) and eluted with H, DCM, and EtOAc gradients (100% H to 100% EtOAc). Six fractions of 1 L each were obtained, which concentrated under reduced pressure to afford F_1_ (200 mg; H 100%), F_2_ (115 mg; 50:50% H/DCM), F_3_ (1200 mg; 90:10% DCM/EtOAc), F_4_ (187.7 mg 80:20% DCM/EtOAc), F_5_ (155 mg; 70:30% DCM/EtOAc), and F_6_ (137 mg; EtOAc 100%) fractions. Fractions F_1_–F_6_ were tested for antimicrobial activity. Fraction F_3_ (1100 mg) permeated through Sephadex LH-20 (column length 23 cm, i.d. 4 cm; petroleum ether (PE)/MeOH/DCM 2:1:1). Some 21 fractions of 15 mL each were obtained. After TLC comparison (silica gel, PE/EtOAc 8:2) as the mobile phase, detection under UV light and spraying with *p*-anisaldehyde sulfuric acid, the fractions with similar TLC patterns were combined as follows: I (477 mg), II (142 mg), III (98 mg), IV (295 mg), and V (84 mg).

#### 3.1.5. Isolation and Characterization of (R)-2-(4-Methylcyclohex-3-en-1-yl)propan-2-yl (E)-3-(4-hydroxyphenyl)acrylate (**1**)

Separation of the fraction IV (295 mg) using column chromatography on silica gel (column length 40 cm, internal diameter 2 cm; 50 g silica gel 0.063–0.2 mesh, Merck 60); (100% H to 100% EtOAc) provided 200 mg of pure compound **1**: oil; ${\left[\alpha \right]}_{D}^{23}$ + 29 (c = 0.17 g/100 mL, CHCl_3_), ^1^H-NMR (400 MHz, CDCl_3_) δ 1.50 (s, 3H, H-9′), 1.53 (s, 3H, H10′), 1.67 (s, 3H, H-7′), 1.85-1.35 (m, 2H, H-3′), 2.12 (m, 1H, H-4′) 2.16-1.86 (m, 4H, H-2′, H-5′), 5.39 (s, 1H, H-6′), 6.22 (d, *J* = 16 Hz, 1H, H-8), 6.85 (d, *J* = 8Hz, 2H, H-3 and H-5), 7.38 (d, *J* = 8Hz, 2H, H-2 and H-6), 7.51 (d, *J* = 16 Hz, 1H, H-7); ^13^C-NMR (100 MHz, CDCl_3_) δ 23.3 CH_3_, δ 23.4 CH_3_, 23.5 CH_3_, 24.0 CH_2_, 26.5 CH_2_, 30.9 CH_2_, 42.8 CH (C-4′), 85.2 C (C-8′), 115.9 CH ×2 (C-3 and C-5), 117.2 CH (C-8), 120.3 CH (C-6′), 127.0 (C-1), 129.9 CH ×2 (C-2 and C-6), 134.0 (C-1′), 143.8 (C-7), 158.4 (C-4), 167.8 C (C=O), HR-EIMS 323.1618, (calcd for C_19_H_24_O_3_ (M + Na) 323.1618). (See spectra in Supplementary Material).

### 3.2. Synthesis

#### 3.2.1. General Methods

All of the reagents were purchased from Aldrich. Kieselgel 60, 0.040–0.063 mm (Merck, Darmstadt, Germany) was used for the column chromatography. TLC experiments were performed on alumina-backed silica gel 40 F_254_ plates (Merck, Darmstadt, Germany). The plates were illuminated under UV (254 nm) and evaluated in iodine vapour. The melting points were determined on an Optimelt MPA-100 apparatus (SRS, Stanford, CA, USA). The purity of the final compounds was checked using HPLC. Detection wavelengths of 210 and 250 nm were chosen for detection. The purity of individual compounds was determined from the area peaks in the chromatogram of the sample solution in order to ensure >95% purity. All ^1^H-NMR spectra were recorded on a Bruker AM-400 (400 MHz for ^1^H and 101/126 MHz for ^13^C), BrukerBioSpin Corp., Rheinstetten, Germany. Chemical shifts are reported in ppm (δ) against the internal standard, Si(CH_3_)_4_. Easily exchangeable signals were omitted when they were diffused. Signals are designated as follows: s, singlet; d, doublet; dd, doublet of doublets; m, multiplet. The high-resolution mass spectra of compounds 3–5 were measured using a high-performance liquid chromatograph Dionex UltiMate® 3000 (Thermo Scientific, Waltham, MA, USA) coupled with a LTQ Orbitrap XLTM Hybrid Ion Trap-Orbitrap Fourier Transform Mass Spectrometer (Thermo Scientific) with injection into HESI II in the positive mode.

#### 3.2.2. Synthesis of (R)-2-(4-Methylcyclohex-3-en-1-yl)propan-2-yl (E)-3-(4-ethoxyphenyl)acrylate (**2**)

In a Schlenk tube equipped with a magnetic stirrer, *t*-BuOK (0.118 mmol) was added to 2 mL of degassed DMSO under nitrogen atmosphere. After 10 min, when no more solid was present, compound **1** (0.098 mmol) was added. The reaction mixture was stirred for 30 min until the formation of the corresponding ions. The reaction was then quenched by adding excess ethyl iodide (0.196 mmol) to obtain compound **2**. The solution was extracted with ethyl acetate and water. The organic extract was analysed by GC-MS. The reaction was then quenched by adding excess ethyl iodide (0.196 mmol) to obtain a brown oil that was purified by column chromatography on silica gel (column length 40 cm, internal diameter 2 cm; 50 g silica gel (0.063–0.2 mesh, Merck 60) using a solvent gradient (100% PE to 100% EtOAc) to afford 110 mg of pure compound **2**: oil (yield 95%); ${\left[\alpha \right]}_{D}^{23}$ + 23 (c = 0.11g/100 mL, CHCl_3_), ^1^H-NMR (400 MHz, CDCl_3_) δ 1,42 (t, 3H, CH_3_ ethyl), 1.50 (s, 3H, H-9′), 1.53 (s, 3H, H10′), 1.65 (s, 3H, H-7′), 1.85-1.35 (m, 2H, H-3′), 2.12 (m, 1H, H-4′) 2.16-1.86 (m, 4H, H-2′, H-5′), 4.05 (q, 2H, CH_2_ ethyl) 5.39 (s, 1H, H-6′), 6.23 (d, *J* = 16 Hz, 1H, H-8), 6.87 (d, *J* = 8Hz, 2H, H-3, H-5), 7.44 (d *J* = 8Hz, 2H, H-2, H-6), 7.52 (d, *J* = 16 Hz, 1H, H-7); ^13^C-NMR (100 MHz, CDCl_3_) δ 14.7 CH_3_ (ethyl) 23.3 CH_3_, 23.4 CH_3_, 23.5 CH_3_, 24.0 CH_2_, 26.5 CH_2_, 30.9 CH_2_, 42.7 CH (C-4′), 63.5 (s, 2 H, CH_2_ ethyl), 84.9 C (C-8′), 114,7 CH (C-3, C-5), 117.6 CH (C-8), 120.4 CH (C-6′), 127.2 (C-1), 129.6 CH (C-2 and C-6), 134.0 (C-1′), 143.3 (C-7), 160.0 CH (C-4), 168.9 C (C=O), HREIMS 351.1935, (calcd for C_21_H_28_O_3_ (M + Na) 351.1931). (See spectra in Supplementary Material).

#### 3.2.3. General Synthesis of Styrylquinoline Derivatives **3**–**5**

The appropriate quinoline derivative (2.5 mmol) in acetic anhydride with 80% acetic acid was thoroughly mixed with one equivalent aldehyde and heated in an inert gas atmosphere (N_2_) at 130 °C for 18 h. Then, the mixture was evaporated to dryness, and a solid was recrystallized from ethanol. The appropriate styrylquinoline derivative (2.5 mmol) in methanol was thoroughly mixed with 2.5 equivalent K_2_CO_3_ at room temperature for 2 h. Then, concentrated HCl was added, and the resulting precipitate was filtered and washed with H_2_O. The crude product was crystallized from ethanol.

2-[2-(4-Methoxyphenyl)vinyl]quinolin-8-yl acetate (**3**): yield 20%, light brown solid, m.p. 97−100 °C; HPLC purity 95.82%; ^1^H-NMR (400 MHz, DMSO-δ_6_) δ 8.38 (d, *J* = 8.6 Hz, 1H), 7.90–7.82 (m, 2H), 7.78 (d, *J* = 16.3 Hz, 1H), 7.70 (d, *J* = 8.5 Hz, 2H), 7.57–7.47 (m, 2H), 7.30 (d, *J* = 16.3 Hz, 1H), 7.02 (d, *J* = 8.5 Hz, 2H), 3.82 (s, 3H), 2.50 (s, 3H); ^13^C-NMR (126 MHz, DMSO-δ_6_) δ 172.49, 160.23, 154.26, 153.29, 138.60, 136.84, 134.57, 129.58, 129.39, 129.11, 127.97, 127.24, 126.14, 122.09, 121.25, 118.02, 114.87, 111.58, 55.69, 21.52. HR-MS: for C_20_H_18_NO_3_ [M + H]^+^ calculated 320.1281 *m/z*, found 320.1284 *m*/*z*.

2-[2-(4-Acetoxyphenyl)vinyl]quinolin-8-yl acetate (**4**): yield 67%, yellow solid, m.p. 157–159 °C; HPLC purity 95.83%; ^1^H-NMR (400 MHz, CDCl_3_) δ 8.15 (dd, *J* = 8.5, 5.1 Hz, 1H), 7.73–7.60 (m, 5H), 7.55–7.37 (m, 3H), 7.19–7.12 (m, 2H), 2.58 (s, 3H), 2.35 (s, 3H); ^13^C-NMR (101 MHz, CDCl_3_) δ 169.84, 169.33, 155.65, 150.91, 147.38, 140.94, 136.41, 134.25, 133.71, 131.21, 129.10, 128.60, 128.32, 125.75, 125.54, 121.95, 121.68, 120.19, 21.17, 21.03. HR-MS: for C_21_H_18_NO_4_ [M + H]^+^ calculated 348.1230 *m*/*z*, found 348.1239 *m*/*z*.

2-[2-(4-Hydroxyphenyl)vinyl]quinolin-8-ol (**5**): yield 63%, orange solid, m.p. 156–157 °C; HPLC purity 98.38%; ^1^H-NMR (400 MHz, DMSO-δ_6_) δ 11.81 (s, 1H), 10.38 (s, 1H), 8.84 (d, *J* = 8.9 Hz, 1H), 8.42 (d, *J* = 8.9 Hz, 1H), 8.22 (d, *J* = 16.2 Hz, 1H), 7.79 (d, *J* = 16.3 Hz, 1H), 7.67–7.56 (m, 4H), 7.47 (dd, *J* = 6.1, 2.6 Hz, 1H), 6.95 (d, *J* = 8.6 Hz, 2H); ^13^C-NMR (126 MHz, DMSO-δ_6_) δ 161.19, 153.30, 148.85, 144.14, 143.99, 143.72, 130.85, 129.57, 128.20, 126.55, 119.16, 118.74, 116.83, 116.50. HR-MS: for C_17_H_13_NO_2_ [M + H]^+^ calculated 264.1019 *m*/*z*, found 264.1028 *m*/*z*.

6-Hydroxy-*N*-phenylnaphthalene-2-carboxamide (**6**) and 6-hydroxy-*N*-(3-trifluoromethylphenyl)-naphthalene-2-carboxamide (**7**) were synthesized and characterized recently [25].

### 3.3. Antibacterial Activity

For the antibacterial evaluation, methicillin-sensitive *Staphylococcus aureus* ATCC 25923, methicillin-resistant *S. aureus* ATCC 43300, *Escherichia coli* ATCC 25922, and clinical isolates from human patients—coagulase negative *Staphylococcus aureus* 502, *Streptococcus pyogenes*, *Streptococcus agalactiae*, *Escherichia coli* LM2, *Salmonella* sp. and *Klebsiella pneumoniae*—provided by Laboratorio de Microbiologia, Hospital Marcial Quiroga, San Juan, Argentina, were used.

Minimal inhibitory concentration (MIC) values were determined using the broth microdilution method according to the protocols of the Clinical and Laboratory Standards Institute [30]. The assay was executed in Mueller–Hinton broth (MHB), using strains 24 h old. The inoculum employed was 1–5 × 10^5^ CFU/mL. Stock solutions of extracts in DMSO (50 mg/mL) were diluted to give serial 2-fold dilutions that were added to each medium to obtain final concentrations ranging from 1000 to 1 µg/mL. Stock solutions of compounds in DMSO (5 mg/mL) were diluted to give serial 2-fold dilutions that were added to each medium to obtain final concentrations ranging from 50 to 1 µg/mL. The final concentration of DMSO in the assay did not exceed 1%. Cefotaxime Argentia^®^ (Bristol-Myers Squibb, Buenos Aires, Argentina) was included in the assays as a positive control, while MHB and DMSO (1%) were used as negative controls. The plates were incubated for 24 h at 37 °C. Activity was evaluated at 620 nm using a Multiskan™ FC Microplate Photometer (Thermo Fisher Scientific, Vantaa, Finland). The MIC values were defined as the lowest extract concentrations showing no bacterial growth after the incubation time. Tests were done in triplicate.

### 3.4. Conformational and Electronic Study

All calculations were carried out using the Gaussian 09 program (Gaussian, Wallingford, CT, USA) [31]. The search for low-energy conformations on the potential energy surface for compounds 1, 3, 6, and 7 was carried out by using combined *ab initio* (RHF/3-21G) and density functional theory (DFT) (B3LYP/6-31G(d)) calculations. Final DFT geometries were obtained from the geometry optimisation jobs. Minima were characterized through harmonic frequency analysis calculated at DFT level. Correlations effects were included using DFT and the functional of Lee [32] as proposed and parameterized by Becke [33,34] (RB3LYP) and 6-31G (d) basis set. Compound **1** looks like a relatively simple conformational problem, in which three torsional angles (ϕ1, ϕ2, and ϕ3, Figure 1) are main rotations in this molecule. Our calculations predict that the torsional angle ϕ1 possesses only two possible quasi-planar conformations: one of them near to 0°, and the opposite position giving values near to 180°. The energy barrier obtained for this rotation is more than 10 Kcal/mol, suggesting a significantly restricted torsional angle (see Figure S1 in Supplementary Material). It should be noted that the *cis* form possesses about 1 Kcal/mol above the *trans* form; therefore, the conformational analysis of compound **1** considering ϕ1 near to 180° was performed. The orientations of the ring B are mainly described by the dihedral angles ϕ2 and ϕ3, and probably the most comprehensive computational method to evaluate how the local energy minima and the transition states are linked together requires an exploration of the complete potential energy surface (PES) generated by ϕ2 vs. ϕ3. This PES was calculated using a 12 × 12 grid generated by rotating through ϕ2 and ϕ3 in 30° increments from −180 to 180°. At each point, a complete geometry optimization was performed with ϕ2 and ϕ3 frozen at their respective grid values. The graphical presentation of the PES was generated using the program AXUN 5.0 (MathSoft, Cambridge, MA, USA), plotting the total energy values as a dependent variable generated by double scans over independent variables ϕ2 and ϕ3. The optimum coordinates of ϕ2 and ϕ3 for all possible minima were then visually estimated from the level contours diagrams (Figure S2 in Supplementary Material). Five different conformations (conformations 1–5) were obtained for **1**; such low-energy conformers might be well appreciated in this surface. The global minimum (conformer 1) corresponds to a partially extended form (see Table S1 in Supplementary Material). On the other hand, the conformational analysis suggests planar forms as energetically preferred structures for compounds **3**, **6**, and **7**. This is an expected result, considering the structural characteristics of their connecting chains. In compound **3**, it is a highly delocalized system, while in compounds **6** and **7**, it is a peptide bond. The electronic study of compounds **1**, **3**, **6**, and **7** was carried out using molecular electrostatic potentials (MEP) [27]. MEP have been shown to provide reliable information both on the interaction sites of the molecules with point charges and on the comparative reactivities of such sites [28,29]. These MEP were calculated using B3LYP/6-311G (d,p) wave functions from the MOLEKEL 4.0. program (CSCS, Manno, Switzerland) [35].

## 4. Conclusions

Strong antibacterial activities found for some of the extracts studied here fully justify the use of infusions and decoctions of *Annona emarginata* (Schltdl.) H. Rainer in folk medicine for the treatment of some infections. First, it is interesting to remark that the less polar flower extracts (hexane, dichloromethane) showed very strong antibacterial activity against methicillin-sensitive *S. aureus* ATCC 25923 and methicillin-sensitive *S. aureus* ATCC 43300. Additionally, the hexane extract showed activity against *K. pneumoniae*, while the methanol extract from the fruits was also active against the three strains mentioned above. Such activity might be explained at least in part by the content of the new main compound (*R*)-2-(4-methylcyclohex-3-en-1-yl)propan-2-yl (*E*)-3-(4-hydroxyphenyl)acrylate (**1**). This compound showed strong antimicrobial activity against all Gram (+) species tested. The demand for new antibacterial agents and the new trend towards the use of characterized natural extracts or compounds isolated as medicines instead of pure drugs might open an alternative way for the development of new antibacterial agents, trying to avoid the serious problem of resistance to the antibiotics in use today. On the other hand, the molecular calculations allowed for the establishment of structure-activity relationships between the antibacterial compounds reported here, which gives important information about a possible pharmacophoric pattern of these compounds. This information may be of great interest for the search and procurement of new structurally related antibacterial agents.

## Figures and Tables

**Table 1:** Antibacterial activity (MIC (µg/mL)) of extracts of *A. emarginata* (*Annonaceae*).

Extracts		MIC (µg/mL)					
		MSSA	MRSA	*E. coli* ATCC 25922	*E. coli* LM2	*K. pneumoniae*	*Salmonella* sp.
**Stem bark**	**HBE**	>1000	>1000	250	500	>1000	1000
	**DCMBE**	1000	>1000	500	>1000	1000	250
	**EtAcOBE**	500	1000	500	500	>1000	500
	**MeOHBE**	500	500	1000	1000	>1000	1000
**Leaves**	**HLE**	500	250	>1000	>1000	>1000	500
	**DCMLE**	500	500	>1000	1000	1000	500
	**EtAcOLE**	500	500	>1000	>1000	250	500
	**MeOHLE**	>1000	>1000	500	500	500	500
**Flowers**	**HFlE**	125	125	>1000	>1000	250	1000
	**DCMFlE**	62.5	16	>1000	>1000	1000	>1000
	**EtAcOFlE**	250	250	1000	>1000	500	>1000
	**MeOHFlE**	>1000	>1000	1000	1000	>1000	>1000
**Fruits**	**MeOHGFE**	500	500	>1000	>1000	250	>1000
**cefotaxime**		0.5	0.5	1.9	0.5	15	12.5

HBE: hexane bark extract; DCMBE: dichloromethane bark extract; EtOAcBE: ethyl acetate bark extract; MeOHBE: methanol bark extract; HLE: hexane leaf extract; DCMLE: dichloromethane leaf extract; EtOAcLE: ethyl acetate leaf extract; MeOHLE: methanol leaf extract; HFlE: hexane flower extract; DCMFlE: dichloromethane flower extract; EtOAcFlE: ethyl acetate flower extract; MeOHFlE: methanol flower extract; MeOHGFE: methanol global fruit extract.

**Table 2:** Antibacterial activity (MIC (µg/mL)) of fractions F_1_-F_6_ from MeOHGEF.

Extracts		MIC (µg/mL)					
		MSSA	MRSA	*E. coli* ATCC 25922	*E. coli* LM2	*K. pneumoniae*	*Salmonella* sp.
**MeOHGFE**		500	500	>1000	>1000	250	>1000
**MeOHGFE Fractions**	**F_1_**	>1000	>1000	>1000	>1000	>1000	>1000
	**F**_**2**_	500	250	>1000	>1000	>1000	>1000
	**F**_**3**_	500	500	500	>1000	125	>1000
	**F**_**4**_	1000	1000	1000	125	1000	>1000
	**F**_**5**_	500	500	1000	250	125	>1000
	**F**_**6**_	250	500	250	250	>1000	>1000
**cefotaxime**		0.5	0.5	1.9	0.5	15	12.5

MeOHGFE: methanol global extract fruit; F1 (200 mg; hex 100%), F2 (115 mg; 50:50% H:DCM), F3 (1200 mg; 90:10% DCM:EtOAc), F4 (187.7 mg 80:20% DCM:EtOAc), F5 (155 mg; 70:30% DCM:EtOAc), and F6 (137 mg; EtOAc100%).

**Table 3:** Antibacterial activity (MIC (µg/mL)) of subfractions F_I-V_ from F_3_.

Extracts		MIC (µg/mL)								
		MSSA	MRSA	SA	SP	SAG	EC	ELM	KP	Ssp
**MeOHGFE fraction F**_**3**_		500	500	500	250	250	500	>1000	125	>1000
**F**_**3**_ **subfractions**	**Subfraction I**	>250	>250	>250	>250	>250	>250	>250	>250	>250
	**Subfraction II**	>250	>250	>250	>250	>250	250	>250	>250	>250
	**Subfraction III**	>250	>250	>250	>250	>250	>250	250	>250	>250
	**Subfraction IV**	16	16	31.2	16	16	>250	125	250	>250
	**Subfraction V**	62.5	62.5	62.5	62.5	62.5	>250	>250	>250	>250
**cefotaxime**		0.5	0.5	0.5	0.25	0.25	1.9	0.5	15	12.5

MSSA = methicillin-sensitive *Staphylococcus aureus* ATCC 25923, MRSA = methicillin-resistant *Staphylococcus aureus* ATCC 43300, SA = coagulase negative *Staphylococcus aureus* 502, SP = *Streptococcus pyogenes*, SAG = *Streptococcus agalactiae*, EC = *Escherichia coli* ATCC 25922, ELM = *Escherichia coli* LM2, KP = *Klebsiella pneumoniae*, Ssp = *Salmonella* sp.

**Table 4:** Antibacterial activity (MIC [µg/mL]) of compound 1 (isolated from subfraction F_IV_) and compounds **2**–**7** obtained by synthesis.

Comp.	MIC (µg/mL)							
	MSSA	MRSA	SA	SP	SAG	EC	ELM	KP
**1**	4	6.25	3.12	3.12	3.12	>50	50	50
**2**	>50	>50	>50	>50	>50	>50	>50	>50
**3**	50	50	50	50	50	>50	>50	>50
**4**	>50	>50	>50	>50	>50	>50	>50	>50
**5**	50	>50	25	25	50	>50	>50	>50
**6**	25	50	50	50	50	>50	25	>50
**7**	12.5	50	12.5	25	12.5	>50	12.5	>50
**CEF**	0.5	0.5	0.5	0.25	0.25	1.9	0.5	15

MSSA = methicillin-sensitive *Staphylococcus aureus* ATCC 25923, MRSA = methicillin-resistant *Staphylococcus aureus* ATCC 43300, SA = coagulase negative *Staphylococcus aureus* 502, SP = *Streptococcus pyogenes*, SAG = *Streptococcus agalactiae*, EC = *Escherichia coli* ATCC 25922, ELM = *Escherichia coli* LM2, KP = *Klebsiella pneumoniae*, CEF = cefotaxime.

## Supplementary Materials

The Supplementary materials are available online.
