# Supplementary material for: The Antimicrobial Activity of Annona emarginata (Schltdl.) H. Rainer and Most Active Isolated Compounds against Clinically Important Bacteria
Source: Molecules. 2018 May 16;23(5):1187. doi: 10.3390/molecules23051187 (PMC6099495; doi:10.3390/molecules23051187)
Supplement: Supplementary file 1 [file molecules-23-01187-s001.pdf]

Supplementary material

Antimicrobial activity of *Annona emarginata* (Schltdl.) H. Rainer and the most active isolated compound, acting against clinically important bacteria. SAR study of structurally related compounds.

Juan G. Dolab[1], Beatriz Lima[2], Ewelina Spaczynska[3], Jiri Kos[4], Natividad Herrera Cano[2], Gabriela Feresin[2], Alejandro Tapia[2], Francisco Garibbotto[1,5] , Elisa Petenatti[1], Mónica Olivella[1], Robert Musiol[3], Josef Jampilek[4,*], Ricardo D Enriz[1,5*]

[1]*Facultad de Química Bioquímica y Farmacia, Universidad Nacional de San Luis, Chacabuco 917. 5700 San Luis Argentina*
[2]*Instituto de Biotecnología-Instituto de Ciencias Básicas. Universidad Nacional de San Juan, Av. Libertador General San Martín 1109 (O). CP 5400, San Juan, Argentina*
[3]*Institute of Chemistry, University of Silesia,75 Pulku Piechoty 1; 41-500 Chorzow, Poland*
[4]*Department of Pharmaceutical Chemistry, Faculty of Pharmacy, Comenius University, Odbojarov 10, 83232 Bratislava, Slovakia.*
[5]*Instituto Multidisciplinario de Investigaciones Biológicas IMIBIO-CONICET. Chacabuco 917, 5700. San Luis, Argentina*

Table of Contents

^{13}C NMR spectrum of (*R*)-2-(4-methylcyclohex-3-en-1-yl)propan-2-yl (*E*)-3-(4-hydroxyphenyl)acrylate (1) 4

DEPT spectrum of (*R*)-2-(4-methylcyclohex-3-en-1-yl)propan-2-yl (*E*)-3-(4-hydroxyphenyl)acrylate (1) 5

NOESY spectra of (*R*)-2-(4-methylcyclohex-3-en-1-yl)propan-2-yl(*E*)-3-(4-hydroxyphenyl)acrylate (1) 6

Circular Dichroism spectra of (*R*)-2-(4-methylcyclohex-3-en-1-yl)propan-2-yl(*E*)-3-(4-hydroxyphenyl)acrylate (1) 9

^{1}H NMR spectrum of (*R*)-2-(4-methylcyclohex-3-en-1-yl)propan-2-yl (*E*)-3-(4-ethoxyphenyl) acrylate (2) 10

^{1}H NMR spectrum of (*R*)-2-(4-methylcyclohex-3-en-1-yl)propan-2-yl (*E*)-3-(4-ethoxyphenyl) acrylate (2) 11

^{13}C NMR spectrum of (*R*)-2-(4-methylcyclohex-3-en-1-yl)propan-2-yl (*E*)-3-(4-ethoxyphenyl) acrylate (2) 12

Circular Dichroism spectra of (*R*)-2-(4-methylcyclohex-3-en-1-yl)propan-2-yl (*E*)-3-(4-ethoxyphenyl) acrylate (2) 14

Figure 1S. Potential Energy Curve (PEC) obtained for torsional angle ϕ 1 of compound 1. PEC was calculated at B3LYP/6-31G (d) level of theory. 15

Figure 2S. Contour graphic of Potential Energy Surface (PES) obtained for compound 1 from RHF/3-21G calculations. Full cycle of rotation (from 0° to 360°) is shown for variables ϕ 2 vs ϕ3. The iso-energy curves included in an energy window of 4 Kcal/mol are denoted in red. 16

Table 1S. Different conformers obtained for compound 1. Torsional angles corresponds to those shown in Figure 1. 17

^{1}H NMR spectrum of (*R*)-2-(4-methylcyclohex-3-en-1-yl)propan-2-yl (*E*)-3-(4-hydroxyphenyl)acrylate (**1**)

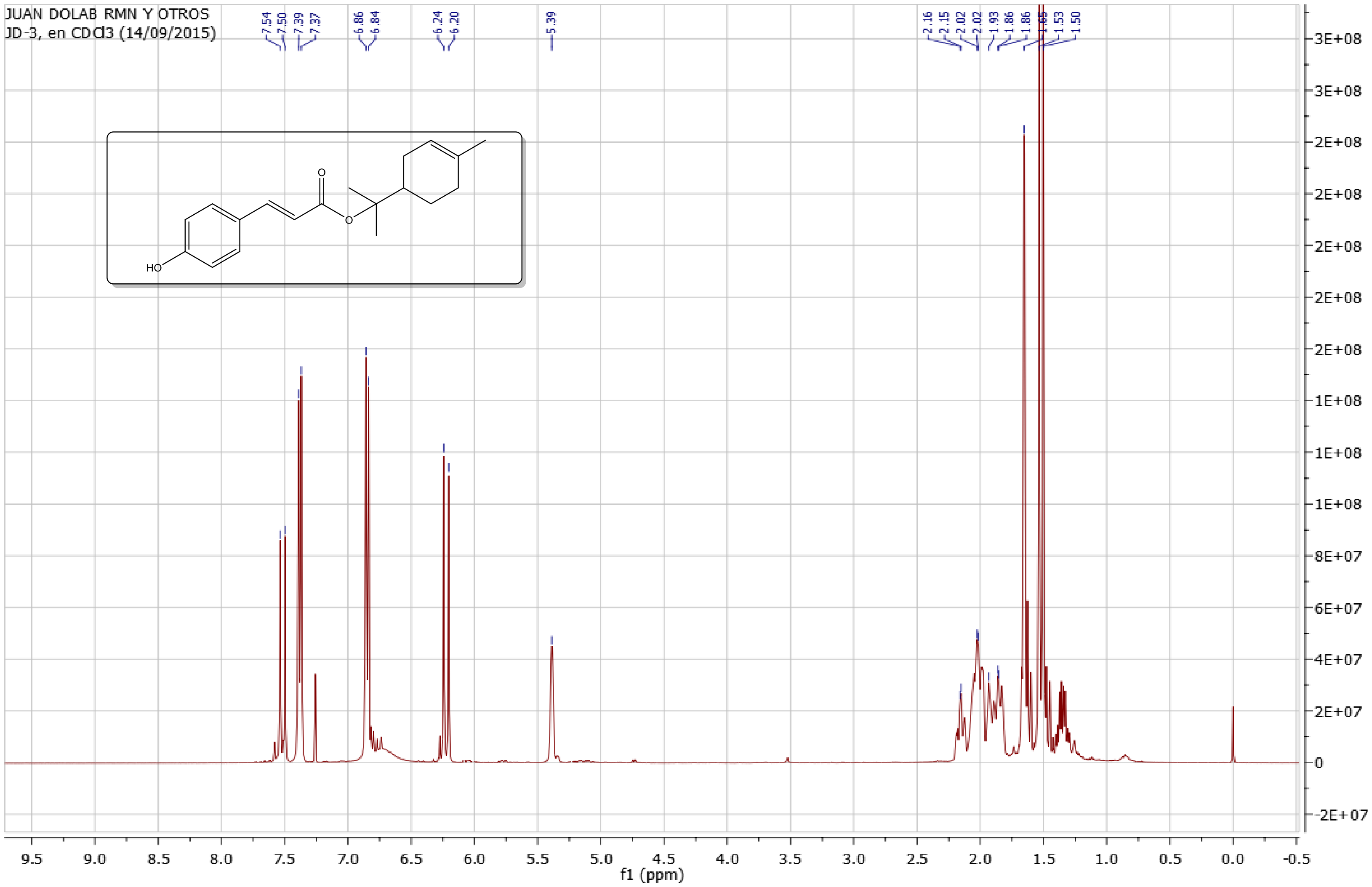

^{13}C NMR spectrum of (*R*)-2-(4-methylcyclohex-3-en-1-yl)propan-2-yl (*E*)-3-(4-hydroxyphenyl)acrylate (**1**)

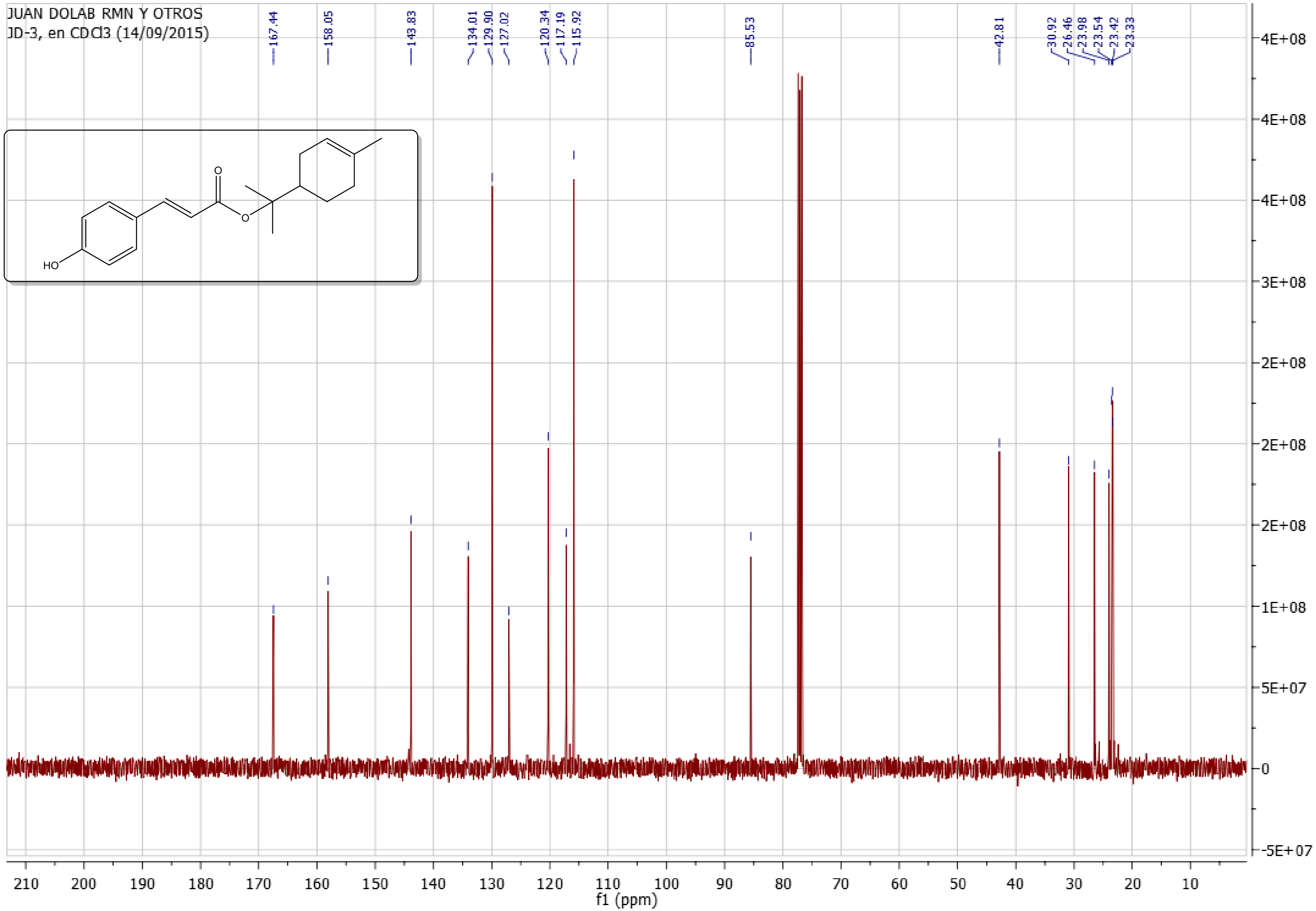

DEPT spectrum of (*R*)-2-(4-methylcyclohex-3-en-1-yl)propan-2-yl (*E*)-3-(4-hydroxyphenyl)acrylate (**1**)

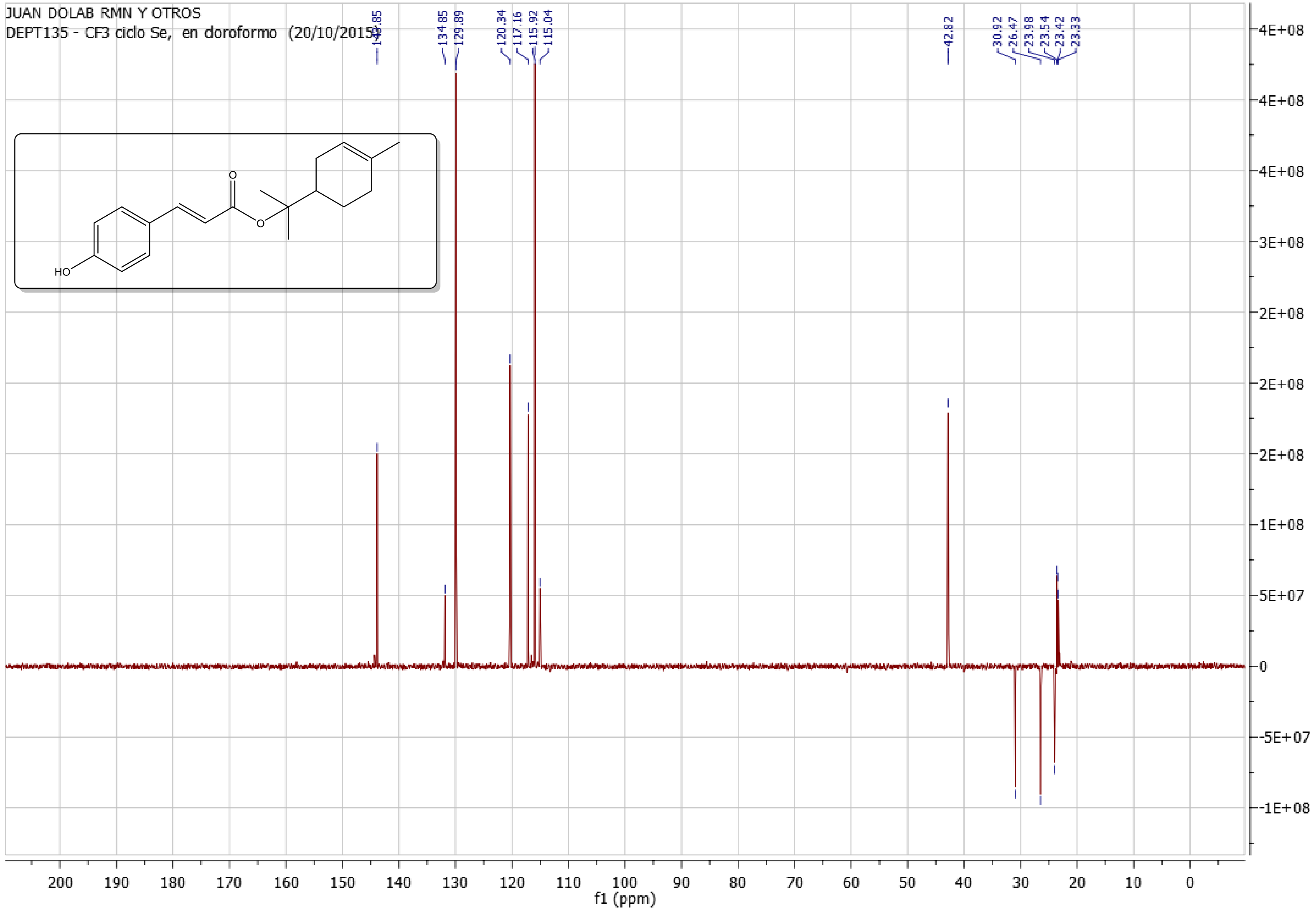

NOESY spectra of (*R*)-2-(4-methylcyclohex-3-en-1-yl)propan-2-yl(*E*)-3-(4-hydroxyphenyl)acrylate (**1**)

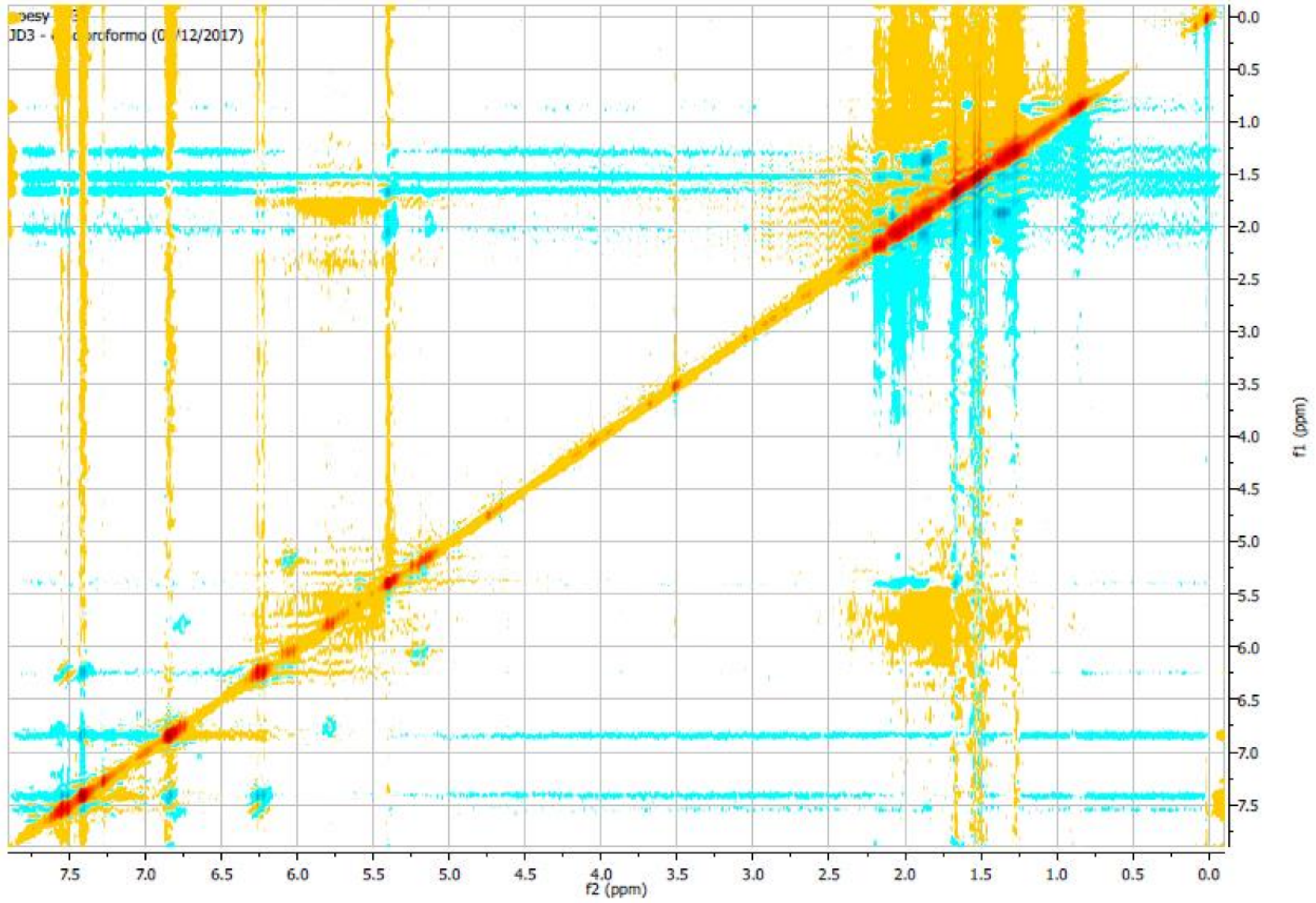

NOESY spectra of (*R*)-2-(4-methylcyclohex-3-en-1-yl)propan-2-yl (*E*)-3-(4-hydroxyphenyl)acrylate (**1**)

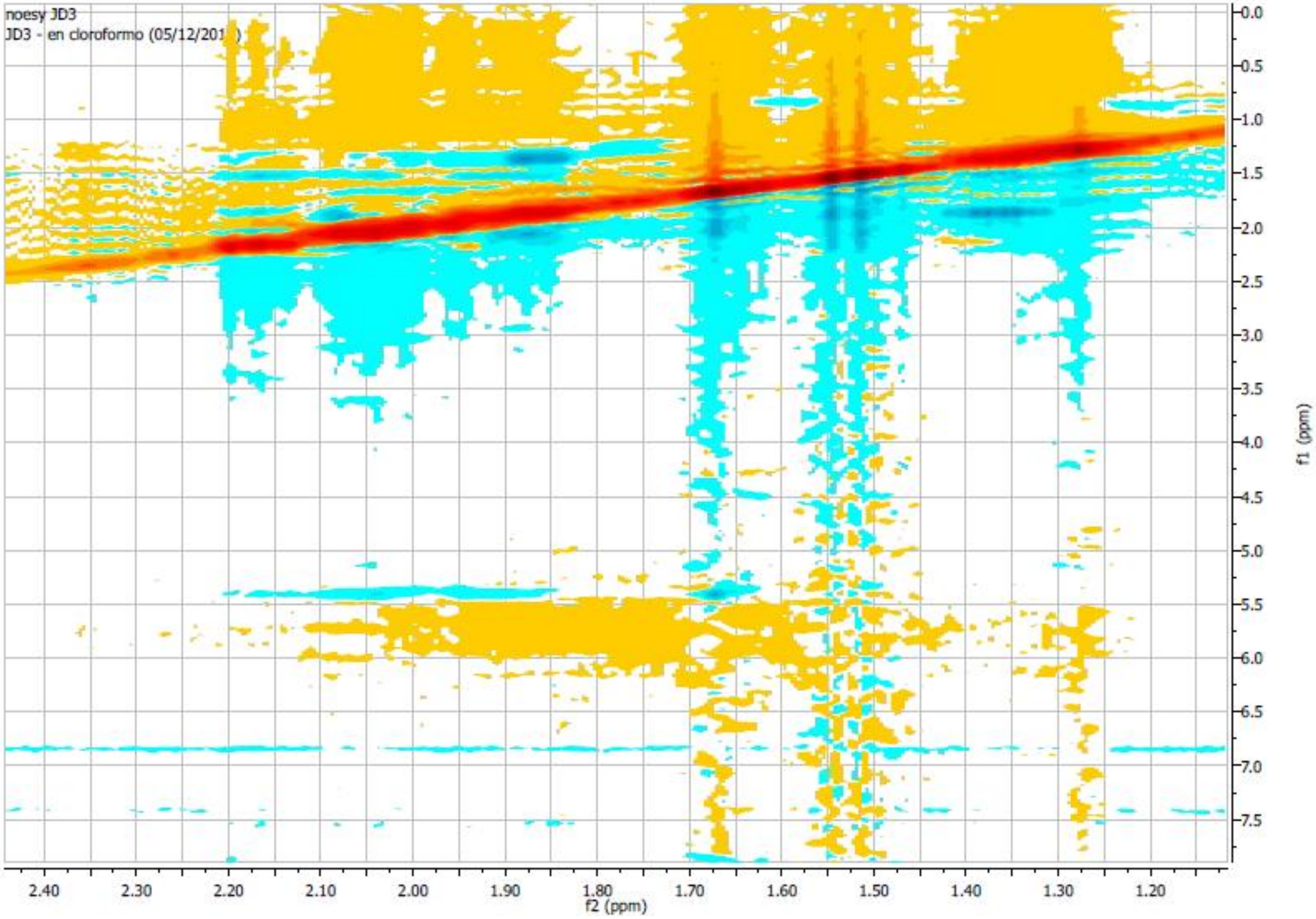

HRMS specrtum of (*R*)-2-(4-methylcyclohex-3-en-1-yl)propan-2-yl(*E*)-3-(4-hydroxyphenyl)acrylate (**1**)

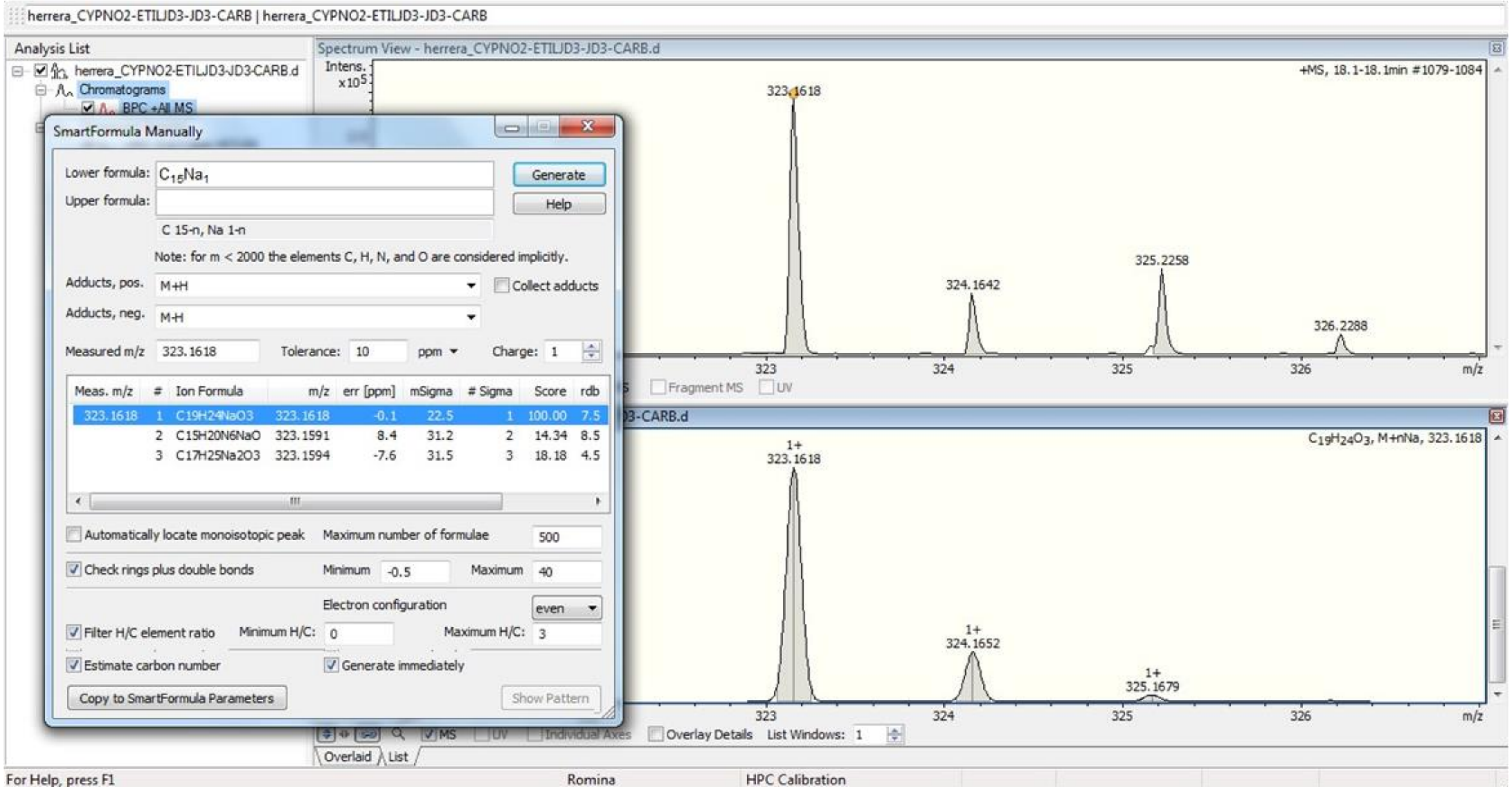

Circular Dichroism spectra of (*R*)-2-(4-methylcyclohex-3-en-1-yl)propan-2-yl(*E*)-3-(4-hydroxyphenyl)acrylate (**1**)

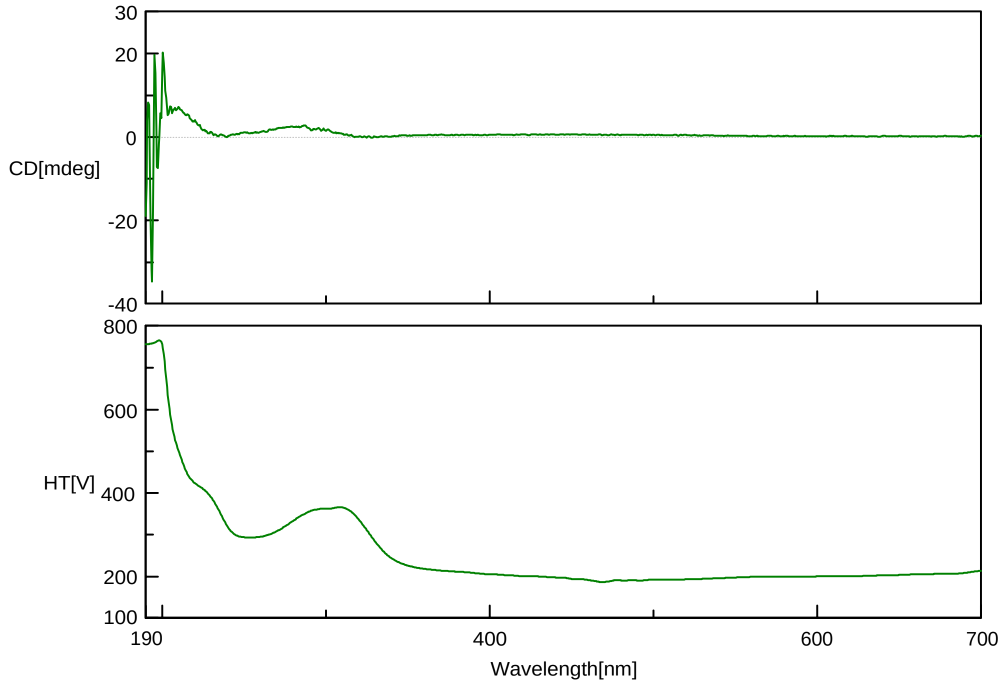

Date	04/12/2017 3:19p.m.
File name	jd3b-blancometanol 2
Model	J-810
Serial No.	
Band width	1 nm
Response	1 sec
Sensitivity	Standard
Measurement range	700 - 190 nm
Data pitch	0.5nm
Scanning speed	100 nm/min
Accumulation	3
Sample name	cata fer
Operator	DC
Comment	

^{1}H NMR spectrum of (*R*)-2-(4-methylcyclohex-3-en-1-yl)propan-2-yl (*E*)-3-(4-ethoxyphenyl) acrylate (**2**)

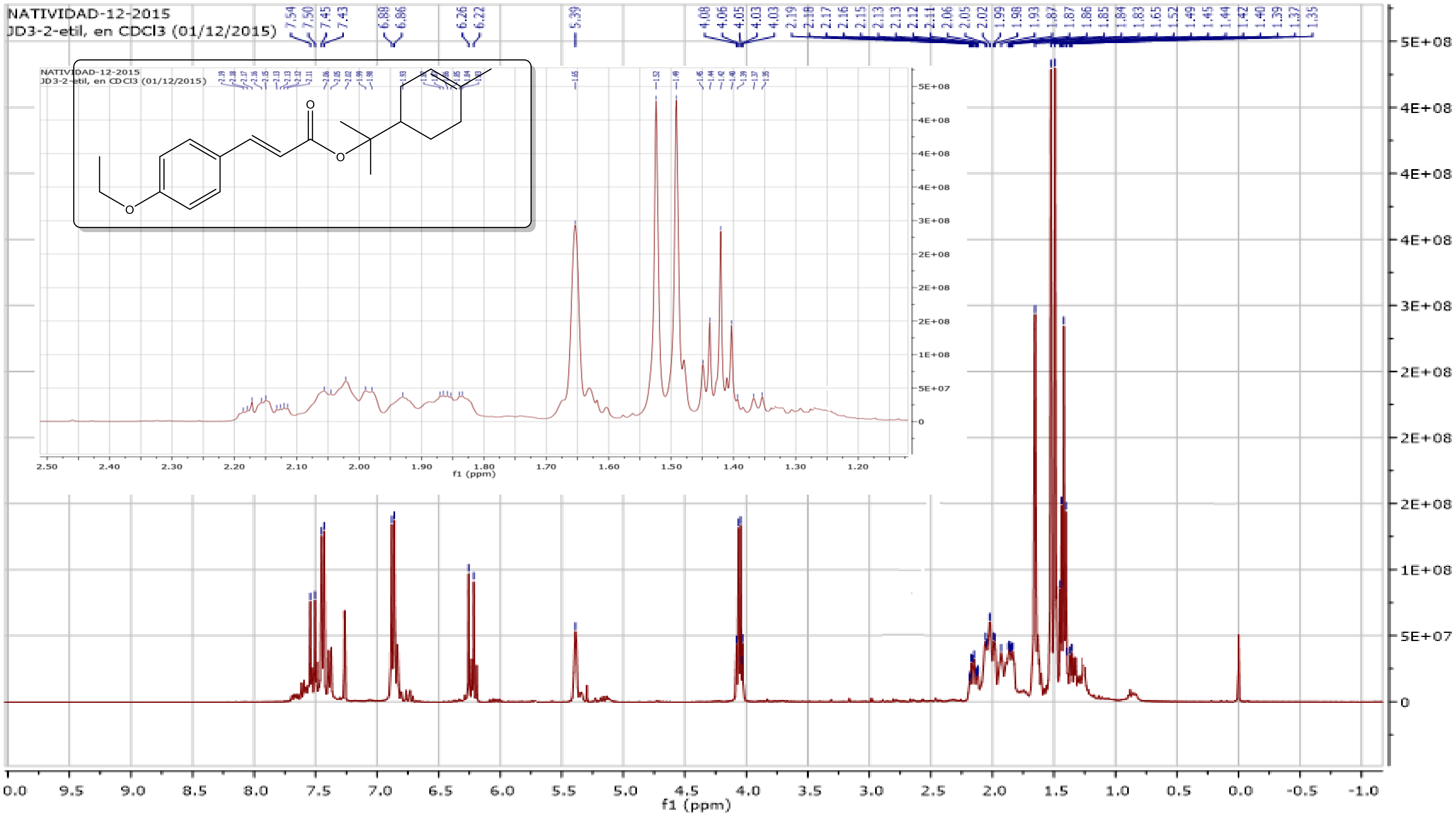

^{1}H NMR spectrum of (*R*)-2-(4-methylcyclohex-3-en-1-yl)propan-2-yl (*E*)-3-(4-ethoxyphenyl) acrylate (**2**)

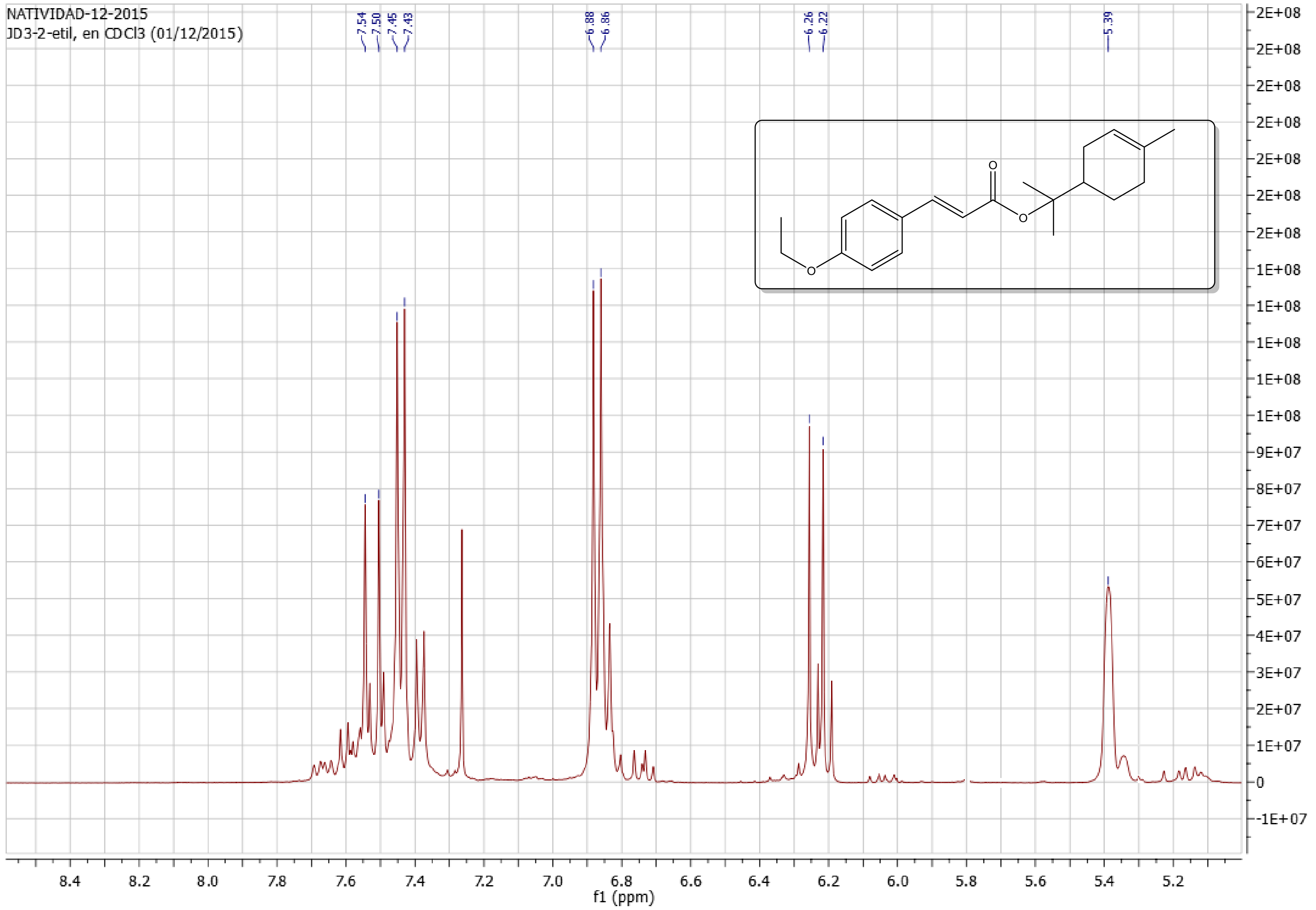

^{13}C NMR spectrum of (*R*)-2-(4-methylcyclohex-3-en-1-yl)propan-2-yl (*E*)-3-(4-ethoxyphenyl) acrylate (**2**)

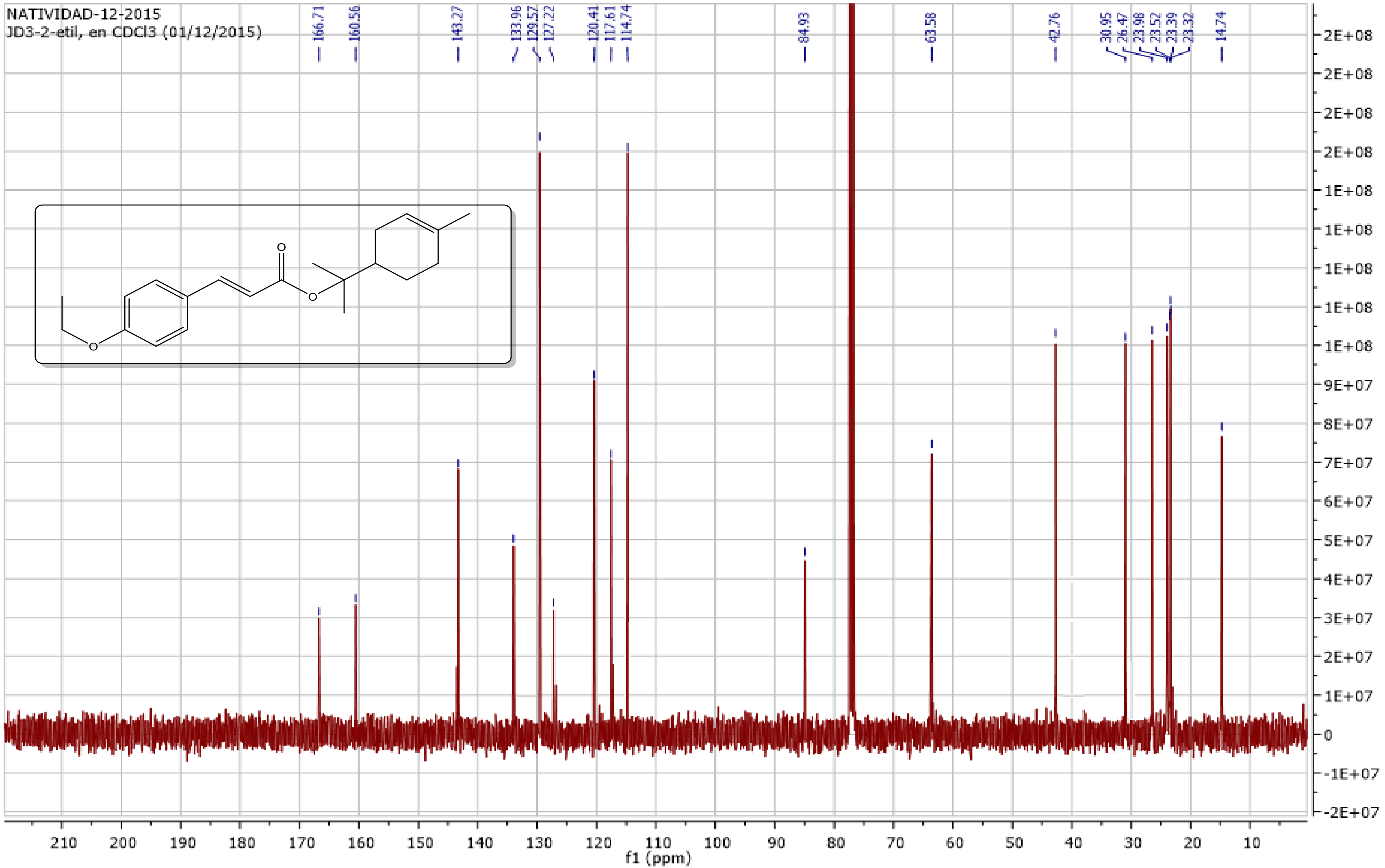

HRMS spectrum of (*R*)-2-(4-methylcyclohex-3-en-1-yl)propan-2-yl (*E*)-3-(4-ethoxyphenyl) acrylate (**2**)

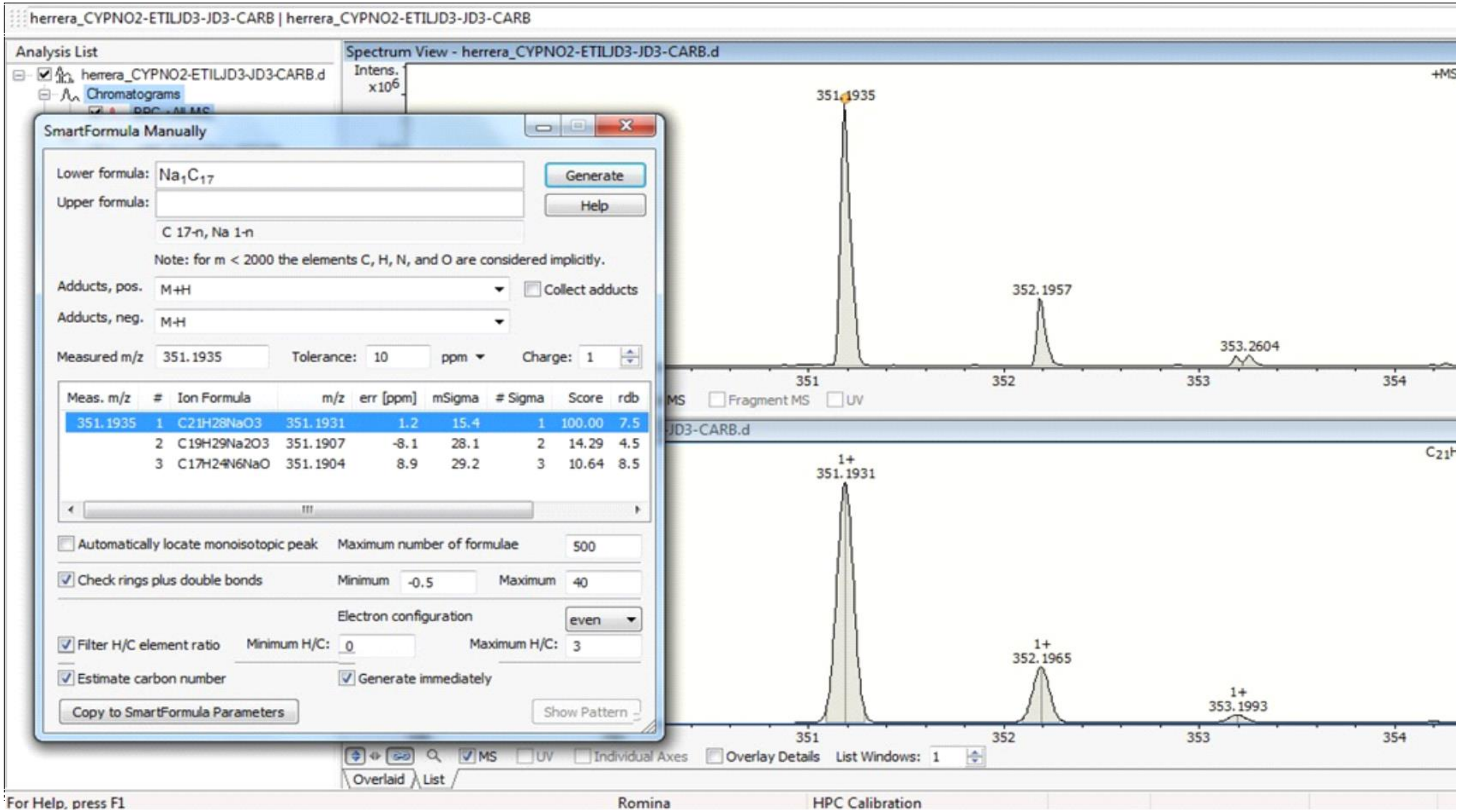

Circular Dichroism spectra of (*R*)-2-(4-methylcyclohex-3-en-1-yl)propan-2-yl (*E*)-3-(4-ethoxyphenyl) acrylate (**2**)

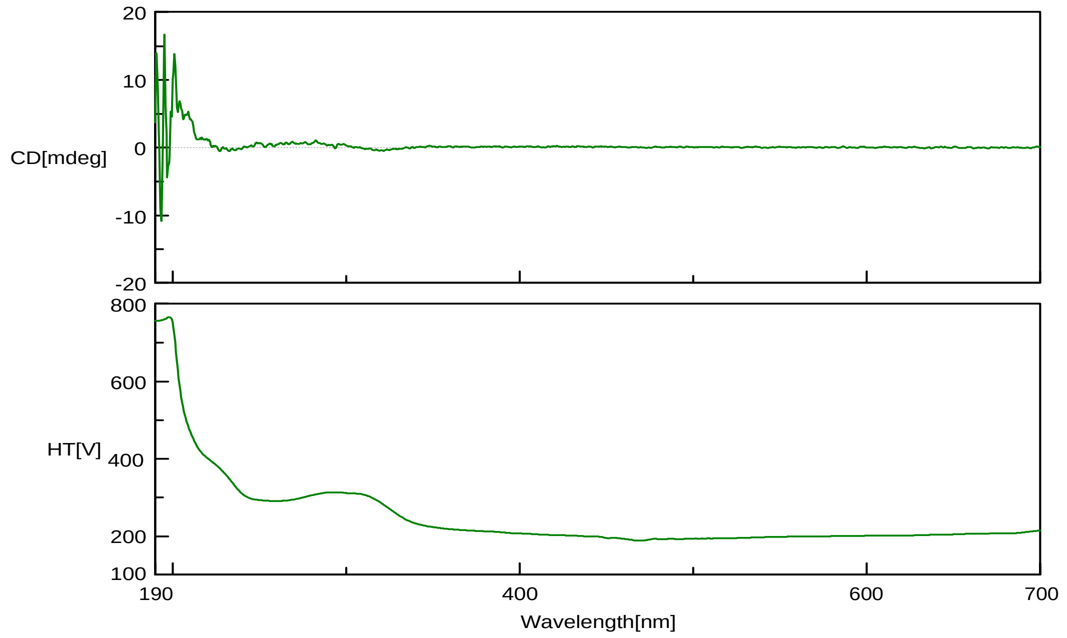

Date	04/12/2017 3:38p.m.
File name	etiljd3-bcometanol 2
Model	J-810
Serial No.	
Band width	1 nm
Response	1 sec
Sensitivity	Standard
Measurement range	700 - 190 nm
Data pitch	0.5nm
Scanning speed	100 nm/min
Accumulation	3
Sample name	cata fer
Operator	DC
Comment	

Figure 1S. Potential Energy Curve (PEC) obtained for torsional angle ϕ 1 of compound 1. PEC was calculated at B3LYP/6-31G (d) level of theory.

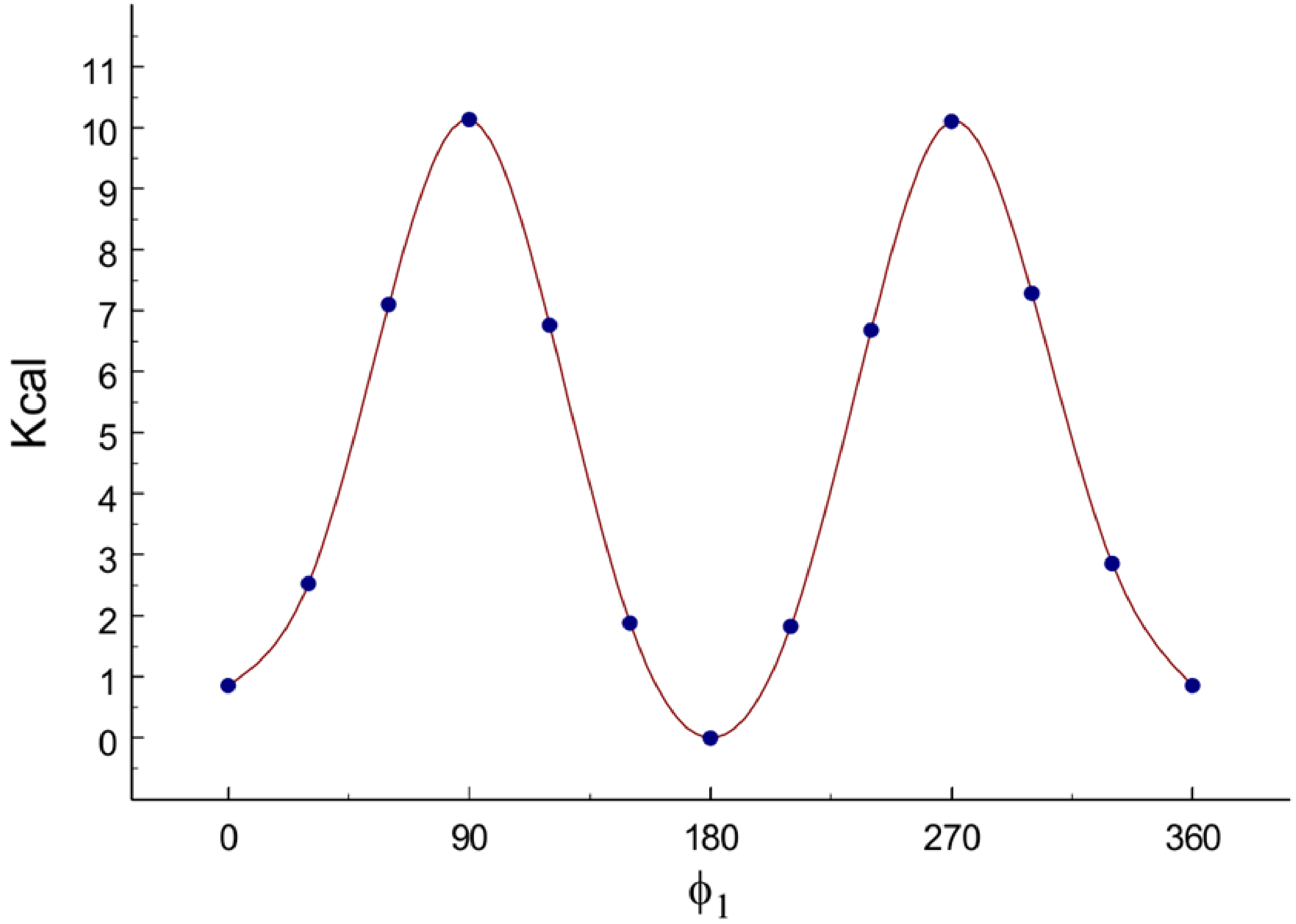

Figure 2S. Contour graphic of Potential Energy Surface (PES) obtained for compound 1 from RHF/3-21G calculations. Full cycle of rotation (from 0° to 360°) is shown for variables ϕ 2 vs ϕ3. The iso-energy curves included in an energy window of 4 Kcal/mol are denoted in red.

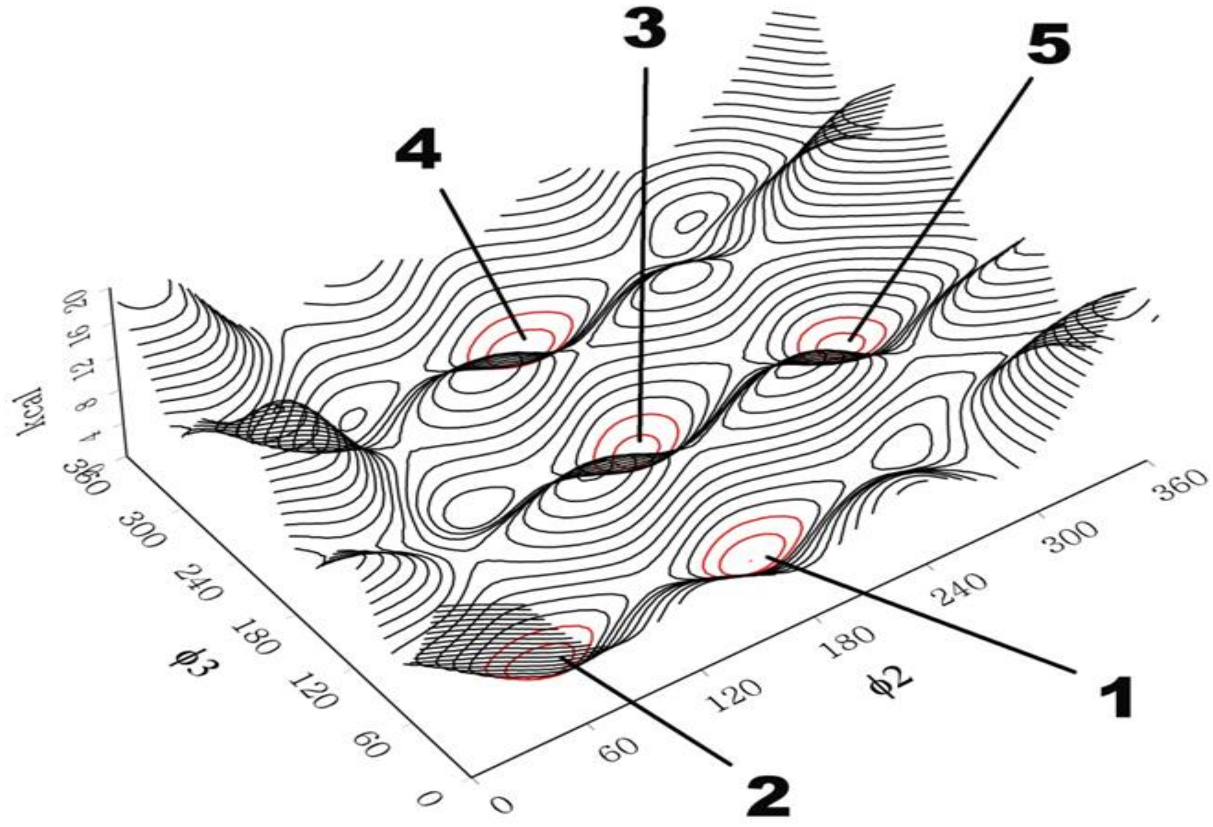

Table 1S. Different conformers obtained for compound **1**. Torsional angles corresponds to those shown in Figure 1.

**Conformer**	**Φ_1**	**Φ_2**	**Φ_3**	**ΔKcal/mol**
1	179,57	-179,9	63,72	0
2	179,6	65,31	57,42	0,16140812
3	-179,9	179,97	179,67	0,45619349
4	178,95	179,34	-60,173	0,02813127
5	-179,86	-63,13	-170,68	0,37493095
